# Supplementary material for: The link between the atherogenic index of plasma and the risk of hypertension: Analysis from NHANES 2017–2020
Source: PLoS One. 2025 Jan 27;20(1):e0317116. doi: 10.1371/journal.pone.0317116 (PMC11771876; doi:10.1371/journal.pone.0317116)
Supplement: S1 Table — (DOCX) [file pone.0317116.s001.docx]

**S1 Table. Logistic analyses between AIP and hypertension after excluding participants younger than 40**

|  | Model1 | | Model2 | | Model3 | |
| --- | --- | --- | --- | --- | --- | --- |
|  | OR (95%CI) | P | OR (95%CI) | P | OR (95%CI) | P |
| AIP | 1.69 (1.41-2.02) | <.001 | 2.70 (1.47-4.95) | 0.001 | 2.13 (1.17-3.90) | 0.014 |
| SBP | 1.03 (1.03-1.03) | <.001 | 1.02 (1.01-1.02) | <.001 | 1.02 (1.01-1.02) | <.001 |
| DBP | 1.02 (1.01-1.02) | <.001 | 1.01 (1.01-1.02) | 0.009 | 1.01 (1.01-1.02) | <.001 |
| HBA1C | 1.32 (1.25-1.39) | <.001 | 1.16 (1.06-1.27) | 0.001 | 1.01 (0.91-1.11) | 0.906 |
| HDL-C | 0.75 (0.65-0.85) | <.001 | 1.83 (1.33-2.51) | <.001 | 1.66 (1.21-2.29) | 0.002 |
| LDL-C | 0.71 (0.65-0.78) | <.001 | 1.05 (0.92-1.20) | 0.488 | 1.07 (0.93-1.22) | 0.345 |
| TC | 0.77 (0.73-0.81) | <.001 | 0.71 (0.65-0.79) | <.001 | 0.77 (0.69-0.84) | <.001 |
| TG | 1.08 (1.02-1.13) | 0.004 | 0.96 (0.86-1.08) | 0.532 | 0.98 (0.88-1.09) | 0.688 |
| BMI | 1.06 (1.05-1.07) | <.001 | 1.05 (1.04-1.07) | <.001 | 1.05 (1.04-1.06) | <.001 |
| ALT | 1.00 (1.00-1.00) | 0.723 | 1.00 (0.99-1.01) | 0.730 | 1.00 (1.00-1.01) | 0.491 |
| AST | 1.00 (1.00-1.00) | 0.770 | 1.00 (0.99-1.01) | 0.994 | 1.00 (0.99-1.01) | 0.780 |
| Scr | 1.01 (1.01-1.01) | <.001 | 1.01 (1.01-1.01) | <.001 | 1.01 (1.01-1.01) | 0.001 |
| FBG | 1.12 (1.09-1.15) | <.001 | 1.02 (0.97-1.06) | 0.524 | 1.01 (0.97-1.06) | 0.581 |
| UA | 1.01 (1.01-1.01) | <.001 | 1.01 (1.01-1.01) | <.001 | 1.01 (1.01-1.01) | <.001 |
| Age | 1.05 (1.04-1.05) | <.001 | 1.04 (1.04-1.05) | <.001 | 1.04 (1.03-1.05) | <.001 |
| SBP: systolic blood pressure; DBP: diastolic blood pressure; LDL-C: low-density lipoprotein cholesterol; TC: total cholesterol; TG: triglycerides; HDL-C: high-density lipoprotein cholesterol; BMI: body mass index ; ALT: alanine aminotransferase ; AST: aspartate aminotransferase; Scr: serum creatinine; AIP: atherogenic index of plasma ; UA: uric acid; FBG :fasting blood sugar; OR: odds ratio; CI: confidence intervals. | | | | | | |
