# Supplementary material for: The link between the atherogenic index of plasma and the risk of hypertension: Analysis from NHANES 2017–2020
Source: PLoS One. 2025 Jan 27;20(1):e0317116. doi: 10.1371/journal.pone.0317116 (PMC11771876; doi:10.1371/journal.pone.0317116)
Supplement: Attachment — Submitted filename: Response to Reviewers.docx [file pone.0317116.s003.docx]

Dear Editors and Reviewers:

  Thank you for your letter and for the reviewers’ comments concerning our manuscript entitled " **The link between the atherogenic index of plasma and the risk of hypertension：Analysis from NHANES 2017–2020**". Those comments are all valuable and very helpful for revising and improving our paper. We have studied comments carefully and have made correction which we hope meet with approval. Revised portion are marked in red in the paper. The main corrections in the paper and the responds to the reviewer’s comments are as follows:

Responds to the reviewer’s comments:

Reviewer #1: The discussion though well written, is not comprehensive enough. The authors main reason to undertake the analysis was to help in resolving previous conflicting results regarding the use of atherogenic index of plasma in predicting hypertension. While the authors have done a good job in writing the discussion, they did not discuss possible reasons for conflicting results. The authors should highlight and discuss any methodological differences between studies with conflicting results and theirs, to bring to light any possible reason for the conflicting results.

Response: We are very sorry for our negligence of the discussion of conflicting results, we have made correction according to the Reviewer’s comments. We have discussed the characteristics of these studies and the potential reasons for the inconsistent results in lines 173 to 185.

Reviewer #2: The topic is highly novel, and the methodology is accurately implemented. However, the author needs to clarify and elaborate on the rationale behind stating that AIP is a good predictor of hypertension, given the sensitivity and specificity values of 53.3% and 58.3%, respectively. Additionally, the AUC of 65.2% does not strongly support AIP as an effective predictor of hypertension.

Response: As Reviewer suggested that the sensitivity and specificity values of 53.3% and 58.3%, respectively. Additionally, the AUC of 65.2% does not strongly support AIP as an effective predictor of hypertension. But our study boasts a relatively large sample size. Additionally, we conducted subgroup analyses and sensitivity analyses to further validate the relationship between AIP and hypertension.

We tried our best to improve the manuscript and made some changes in the manuscript.  And here we did not list the changes but marked in red in revised paper. We appreciate for Editors/Reviewers’ warm work earnestly, and hope that the correction will meet with approval. Once again, thank you very much for your comments and suggestions.

Best regards!

Yours sincerely, Kaiyou Liu

Corresponding author: Kaiyou Liu

E-mail: lkyqrm@163.com
